# Single center analysis of an advisable control interval for follow-up of patients with PI-RADS category 3 in multiparametric MRI of the prostate

**DOI:** 10.1038/s41598-022-10859-9

**Published:** 2022-04-25

**Authors:** M. Boschheidgen, L. Schimmöller, S. Doerfler, R. Al-Monajjed, J. Morawitz, F. Ziayee, D. Mally, M. Quentin, C. Arsov, P. Albers, G. Antoch, T. Ullrich

**Affiliations:** 1 Department of Diagnostic and Interventional Radiology, Medical Faculty, University Dusseldorf, 40225 Düsseldorf, Germany; 2 Department of Urology, Medical Faculty, University Dusseldorf, 40225 Düsseldorf, Germany

**Keywords:** Medical research, Cancer

## Abstract

To evaluate if follow-up mpMRI scans of patients in PI-RADS category 3 are safe enough to omit or delay prostate biopsy in the future and to determine an optimal control interval. This retrospective single center study includes consecutive PI-RADS category 3 patients with one or more follow-up mpMRI (T2WI, DWI, DCE) and subsequent MRI-targeted and systematic TRUS-guided biopsy between 2012 and 2018. Primary study objective was the verification of a significant PI-RADS category upgrade in follow-up mpMRI in patients with subsequent PCA positive biopsy versus patients with negative biopsy. Further objectives were development of the PI-RADS category and clinical parameters between initial and follow-up mpMRI in the context of histopathologic results and time interval. Eighty-nine patients (median PSA 6.6 ng/ml; PSAD 0.13 ng/ml/ml) were finally included (follow-up period 31 ± 18 months). 19 cases had PCA (median PSA 7.8 ng/ml; PSAD 0.14 ng/ml/ml). 4 cases had csPCA (median PSA 5.4 ng/ml; PSAD 0.13 ng/ml/ml) for which there was a significant PI-RADS upgrade after 12–24 months (mean 3.75; p = 0.01) compared to patients without PCA (mean 2.74). Without PCA the mean PI-RADS category decreased after 25–36 months (mean 2.74; p = 0.02). Clinical parameters did not change significantly except a PSAD increase for PCA patients after 24 months. Patients within PI-RADS category 3 may not need prompt biopsy since those with PCA reliably demonstrate a PI-RADS category upgrade in follow-up mpMRI after 12–24 months. PI-RADS 3 patients with negative biopsy do not benefit from follow-up mpMRI earlier than 24 months.

## Introduction

MpMRI of the prostate has meanwhile become the gold standard of prostate cancer diagnostics providing high sensitivity in cancer detection on the one hand and a high negative predictive value to exclude clinically significant prostate cancer on the other hand, thus helping to reduce overtreatment^1–8^. Nevertheless, cancer detection rates of the PI-RADS can vary widely among different institutions or readers expertise and there is ongoing controversy of whether or not equivocal PI-RADS 3 lesions require early biopsy or not^3,9^. Indistinct changes induced by chronic inflammation or (atypical) stromal hyperplasia additionally hamper definite cancer visualization, especially in unclear cases^10,11^. The British NICE guideline and the European EAU guideline still recommend biopsy in PI-RADS 3 lesions despite the well-known disadvantages of overdiagnosis and overtreatment^12^. Specific management and follow-up recommendations for these equivocal PI-RADS lesions instead of biopsy do not exist. Another unsolved problem is the analysis of the optimal time interval between the initial and follow-up mpMRI, either with or without conducted biopsy, reaching from a few months up to several years^13^. Some studies suggest PSAD values in addition to the PI-RADS category to trigger or to delay biopsy^14,15^. The current version of PI-RADS v2.1 does not incorporate management recommendations and does also not provide guiding on evaluation of serial mpMRI.

In this study we analyzed follow-up mpMRI scans of patients with initial PI-RADS category 3 including the development of the PI-RADS category and clinical parameters over time. The aim was to verify that those patients in PI-RADS category 3 who harbour or develop PCA can be detected via PI-RADS upgrade in the follow-up mpMRI. Also, we strived to evaluate an optimal time interval for follow-up MRI scans for PI-RADS 3 cases.

## Material and methods

### Study design

**Figure 1:**
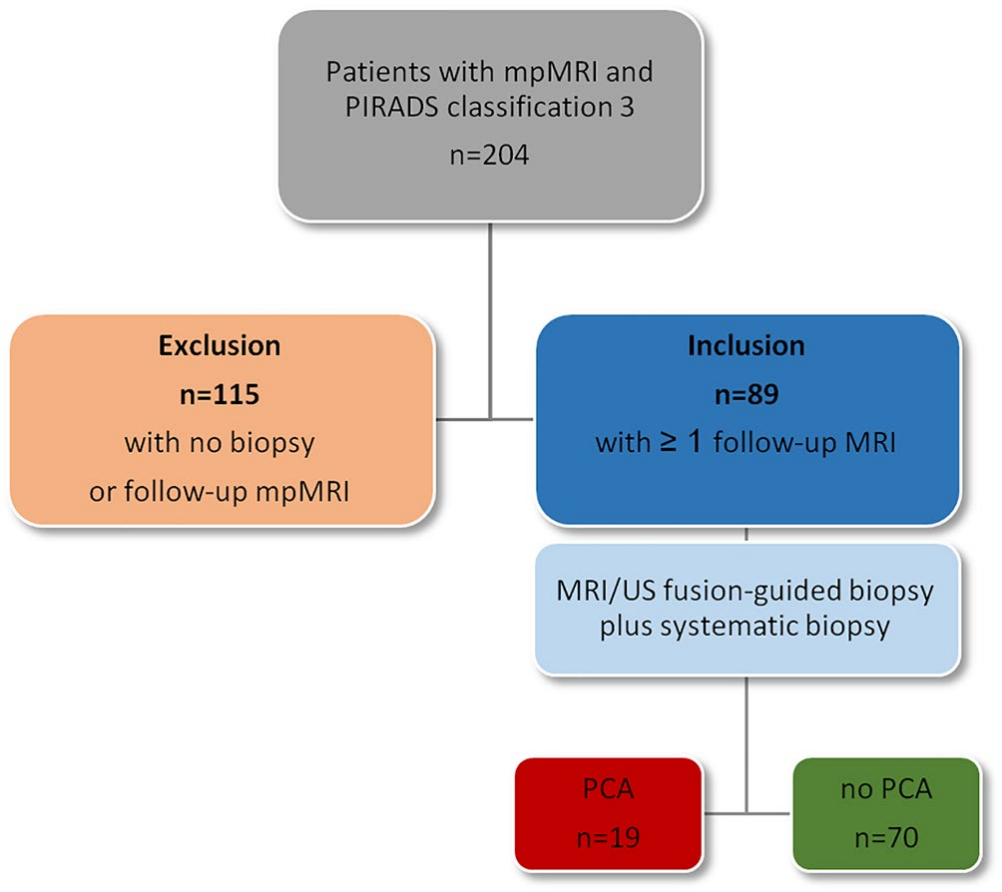
Flowchart of included patient. mpMRI: multiparametric magnetic resonance imaging; PI-RADS: Prostate Imaging Reporting and Data System; PCA: prostate cancer.

This retrospective single center study includes consecutive patients with initial PI-RADS category 3 and one or more follow-up mpMRI (T2WI, DWI, DCE) and subsequent MRI-targeted and systematic TRUS-guided biopsy after follow-up mpMRI between 2012 and 2018. The study was approved by the Ethics Committee, Faculty of Medicine, Heinrich-Heine University of Duesseldorf, Germany. All experiments were performed in accordance with relevant guidelines and regulations**.** Informed consent was obtained from all subjects for the present study**.** Patients without biopsy or without follow-up mpMRI where excluded (Fig. 1). PI-RADS categories and clinical parameters (PSA, PSAD, prostate volume) between initial and follow-up mpMRI and between patients with or without PCA in subsequent biopsies were compared. Additional subgroup analysis of different time intervals between initial and follow-up examinations sought to determine the optimal time interval for control scans. PI-RADS scoring at baseline and follow-up was performed without knowledge of the histopathologic results at the time of the MRI by the same two readers (T.U., L.S.) with at least 6 years’ experience in prostate MRI. In ambigous or difficult cases, decision was made in consensus between the two readers.

### Study objectives

Primary study objective was the verification of a significant PI-RADS category upgrade in follow-up mpMRI in patients with subsequent PCA positive biopsy versus patients with negative biopsy. Further objectives were development of the PI-RADS category and clinical parameters between initial and follow-up mpMRI in the context of histopathologic results and depending on the time interval (12, 24, 36 months).

### Imaging acquisition

All scans were conducted on 3 T MRI scanners (Magnetom TIM Trio, Prisma, or Skyra; Siemens Healthcare GmbH) using either an 18-channel phased-array surface coil combined with a 32-channel spine coil or a 60-channel phased-array surface coil. MR imaging parameters were chosen according to international recommendations and PI-RADS v2.1 guidelines and contained T2-weighted sequences in 3 planes (T2WI; turbo spin echo, TSE; axial: voxel size 0.5 × 0.5 × 3.0 mm; FOV 130 mm), diffusion-weighted imaging (DWI; ss-EPI and rs-EPI; voxel size 0.9–1.4 × 0.9–1.4 × 3.0 mm; b values 0, 500, 1000 s/mm^2^ and 1800 s/mm^2^), and dynamic contrast-enhanced imaging (DCE; T1 vibe; voxel size 0.8–1.5 × 0.8–1.5 × 3.0 mm, scan time 3 min, temporal resolution 7 s). Apparent diffusion coefficient (ADC) parameter maps and high b-values (1800 s/mm^2^) were calculated by the scanner using the standard monoexponentially model.

### Biopsy and histopathology

Targeted MRI/ultrasound fusion-guided biopsy (two targeted cores from each lesion) and subsequent systematic 12-core TRUS-GB were conducted on an MRI/US fusion-guided biopsy system with elastic registration (UroNAV, Invivo, Gainsville, USA). All biopsies were performed by urologists with at least more than 4 years’ experience in MRI-targeted transrectal prostate biopsy (D.M., C.A.). Results from a second histopathologic sample were also considered, i.e., re-biopsy during follow-up. All histopathological findings were analyzed based on the recommendations of the International Society of Urological Pathology to distinguish between csPCA (Gleason score ≥ 7; ISUP grade group 2) and nsPCA (Gleason score 6; ISUP grade 1)^16^.

### Statistical analysis

Statistics were performed using IBM SPSS® Statistics (Version 27 IBM Deutschland GmbH). P-values < 0.05 were defined as statistically significant. Descriptive statistics included mean values and standard deviation for normally distributed variables and median and interquartile range for non-parametric data. Wilcoxon signed rank test was used to compare paired data. Friedman test and ANOVA were used to compare paired data of multiple groups. Post-hoc analysis was conducted to evaluate differences between single groups. Kolmogorov–Smirnov-test and Shapiro–Wilk-test were used to check for normal distribution. Levene-test was applied to check data for homogeneity.

## Results

### Study population

Two-hundred four patients with overall PI-RADS category 3 in mpMRI of the prostate were initially enrolled. 89 patients (mean age 59 ± 9 years) had a follow-up examination within 15 months after the first scan and subsequent targeted and systematic biopsy and were finally included (Fig. 1). 70 of 89 patients (79%) had negative results in the histopathology, whereas PCA was detected in 19 patients (21%), including 4 with csPCA (4%) (n = 15 ISUP grade 1, n = 3 ISUP grade 2, n = 1 ISUP grade 3). The median PSA value of the entire study population increased significantly from 6.6 ng/ml (IQR 5.1–8.6 ng/ml) to 8.2 ng/ml (IQR 5.4–11 ng/ml; p < 0.001) between the two time points (Table 1). The prostate volume also increased from 51 ml (IQR 39–66 ml) to 60 ml (IQR 45–86 ml; p < 0.001). The median PSAD and PI-RADS did not change significantly for the entire population between initial and follow-up examination.

**Table 1:** Baseline characteristics initially and during follow up.

		Initial	FU	p-value*
Number	n	89	89	
PSA, ng/ml	Median (IQR)	6.6 (5.1–8.6)	8.2 (5.4–11)	** < 0.001**
PSAD, ng/ml/cm^3^	Median (IQR)	0.13 (0.10–0.17)	0.13 (0.09–0.17)	0.56
Prostate volume, ml	Median (IQR)	51 (39–66)	60 (45–86)	** < 0.001**
PI-RADS	Median (mean)	3 (3.0)	3 (2.89)	0.11

FU: follow up; PSA: prostate specific antigen; PSAD: prostate specific antigen density; PI-RADS: Prostate Imaging Reporting and Data System; IQR: interquartile range.

*Wilcoxon signed rank test.

Significant values are given in bold.

### Comparison of patients with and without PCA/csPCA during follow-up

The given mean overall PI-RADS classification in the follow-up mpMRI differed significantly between the subgroups of patients without PCA, without csPCA, with PCA, and with csPCA in the subsequent histopathologic evaluation, respectively (Table 2; p = 0.01). Post-hoc analysis for single group evaluation revealed statistically higher given PI-RADS categories for the group with csPCA (PI-RADS median 4, mean 3.75) compared to patients without PCA (PI-RADS median 3, mean 2.74; p = 0.009). All patients with biopsy proven csPCA received a PI-RADS upgrade in the follow-up mpMRI. The median PSA value, PSAD, and prostate volume did not differ significantly between the follow-up subgroups. Exact values are illustrated in Table 2.

**Table 2:** Comparison of patients without and with PCA/csPCA.

	FU without PCA	FU without csPCA	FU with PCA	FU with csPCA	p-value*
Number n	70	85	19	4	
PSA Median (IQR), ng/ml	8.4 (5.5–11)	8.4 (5.5–11)	7.8 (5.3–10.6)	5.4 (4.2–7.9)	0.36
PSAD Median (IQR), ng/ml/cm^3^	0.13 (0.09–0.17)	0.13 (0.09–0.17)	0.14 (0.12–0.18)	0.13 (0.12–0.16)	0.92
Volume Median (IQR) ml	68 (47–91)	62 (45–86)	52 (38–65)	44 (32–50)	0.72
PI-RADS Median (mean)	3 (2.74)	3 (2.85)	4 (3.56)	4 (3.75)	**0.01**
ISUP grade group n	1	15	15	0	
	2	0	3	3	
	3	0	1	1	
	4–5	0	0	0	

FU: follow up; PCA: prostate cancer; csPCA: clinically significant prostate cancer; PSA: prostate specific antigen; PSAD: prostate specific antigen density; PI-RADS: Prostate Imaging Reporting and Data System; ISUP: International Society of Urological Pathology; IQR: interquartile range; n: number.

*Wilcoxon signed rank test.

Significant values are given in bold.

### Follow-up of patients without PCA

**Figure 2:**
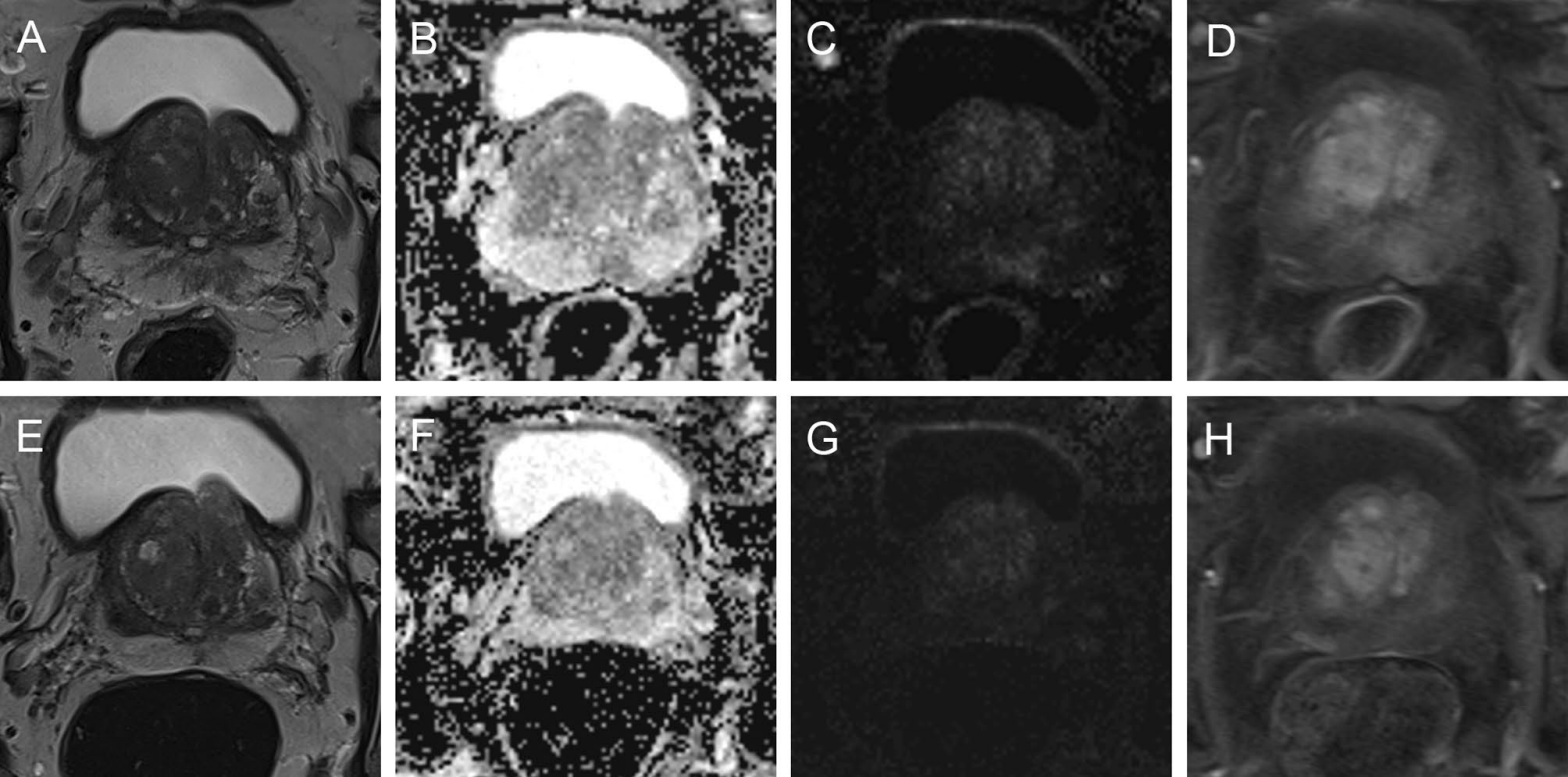
64-year-old patient with rising PSA 12.2 ng/m. Initial MRI examination: Axial T2-weighted image (**A**) showed a non-circumscribed, rounded, moderate hypointensity in the left lateral peripheral zone with focal discrete hypointense ADC signal (**B**), focal discrete hyperintense signal on high b-value DWI (**C**) and discrete correlating contrast enhancement in DCE (**D**), assessed as PI-RADS category 3. Follow-up MRI after 38 months: Axial T2-weighted image (**E**) showed only residual changes, no ADC reduction (**F**), no more hyperintense signal on high b-value DWI (**G**), and no contrast enhancement (**H**). The PSA decreased (6.6 ng/mL) and mpMRI was downgraded to PIRADS category 2.

There was a significant PI-RADS downgrade in cases with negative biopsy over the course of 25–36 months from mean PI-RADS 3.0 to 2.78 (p = 0.02) (Table 3, Fig. 2). The median PSA value, PSAD, and prostate volume did not differ significantly between the different time points among patients without PCA (p = 0.24, 0.06, 0.08, respectively). Exact values are illustrated in Table 3.

**Table 3:** Follow up of patients without PCA.

	Initial	≤ 12 M	12–24 M	25–36 M	p-value
Number n	70	25	46	23	
PSA Median (IQR), ng/ml	8.4 (5.5–11)	7.3 (5.0–10.3)	8.3 (5.7–12.3)	8.5 (7.1–13.4)	0.24
PSAD Median (IQR), ng/ml/cm^3^	0.13 (0.09–0.17)	0.14 (0.11–0.17)	0.11 (0.09–0.21)	0.13 (0.09–0.2)	0.06
Volume Median (IQR), ml	68 (47–91)	53 (39–66)	67 (48–88)	69 (51–80)	0.08
PI-RADS Median (mean)	3 (3.0)	3 (2.84)	3 (2.93)	3 (2.78)	**0.02**
M mean ± SD		10 ± 2	18 ± 3	30 ± 3	

PSA value: prostate specific antigen; PSAD: prostate specific antigen density; PI-RADS: Prostate Imaging Reporting and Data System; IQR: interquartile range; M: months; n: number.

Significant values are given in bold.

### Follow-up of patients with PCA

In the subgroup of patients with proven PCA, the median PSAD increased significantly from 0.13 ng/ml/ml (IQR 0.13–0.18 ng/ml/ml) to 0.18 ng/ml/ml (IQR 0.13–0.22 ng/ml/ml) after 24 months (p < 0.001). Table 4 illustrates the exact values for the distinct time intervals. The mean PI-RADS classification differed significantly between the investigated time intervals (p = 0.02). Post-hoc analysis revealed a significant mean PI-RADS upgrade from 3.0 to 3.88 after 12 -24 months (p = 0.03). The development of PI-RADS in patients with PCA divided by ISUP group is shown in Supplemental Table 1.

**Table 4:** Follow up data of patients with PCA.

	Initial	≤ 12 M	12–24 M	> 24 M	p-value
Number n	19	7	8	10	
PSA Median (IQR) ng/ml	6 (6–9.2)	5.5 (5.4–8.2)	6.9 (5.4–9.1)	8.5 (6.1–11.1)	0.52
PSAD Median (IQR), ng/ml/cm^3^	0.13 (0.13–0.18)	0.12 (0.09–0.16)	0.13 (0.11–0.15)	0.18 (0.13–0.22)	** < 0.001**
Volume Median (IQR), ml	46 (46–65.5)	48 (46–61)	50 (31–66)	57 (38–73)	0.08
PI-RADS Median (mean)	3 (3.0)	3 (3.43)	4 (3.88)	3 (3.30)	**0.02**
M Mean ± SD		9 ± 2	14 ± 2	39 ± 13	

PSA value: prostate specific antigen; PSAD: prostate specific antigen density; PI-RADS: Prostate Imaging Reporting and Data System; IQR: interquartile range; M: months; n: number.

Significant values are given in bold.

## Discussion

Our results suggest that patients with initial PI-RADS category 3 lesions who receive a biopsy positive for PCA over the course of the follow-up, reliably show a PI-RADS upgrade in follow-up mpMRI after 12–24 months. In contrary, patients with PI-RADS 3 lesions without PCA positive biopsies during follow-up, receive a PI-RADS downgrade after 25–36 months. Consequently, follow-up mpMRI of PI-RADS category 3 lesions after 12–24 months seems useful and may justify a delay or even waiver of biopsy.

The overall detection of csPCA in patients with PI-RADS 3 lesions in our collective is very low (4%), which stands in line with previously published data^5,17–20^. We did not observe the detection of PCA with ISUP grade ≥ 3 at all in our patient cohort. The decision whether to proceed to biopsy immediately or perform follow-up MRI after a defined period is discussed controversially. Our findings suggest that follow-up via mpMRI instead of prompt biopsy of these patients does not lead to missed csPCA along the way, a strategy that has been proposed many times by other authors^10,21–23^. The main advantage of follow-up MRI is to avoid unnecessary invasive diagnostic and to ensure higher compliance, as patients often prefer conducting follow-up MRI over biopsy. Besides, information of follow-up MRI is useful to evaluate lesions over time. The comparison to baseline scans can be conducted analogously to the PRECISE criteria in Active Surveillance^24^. That includes the evaluation of ADC-values, size, and contrast enhancement in the course of time. The overall low risk for metastasis in ISUP grade 1 and 2 (< 10%) supports this approach^25^. Nevertheless, standardized management recommendations for PI-RADS 3 patients and especially specific follow-up intervals have not been established yet. The time interval of 12–24 months after which a PI-RADS upgrade was seen in our patients with csPCA has already been reported by a study of Steinkohl at al.^13^. In our cohort all patients with csPCA in subsequent biopsy received a PI-RADS upgrade. However, detection rates are heavily dependent on MRI quality, biopsy, pathology quality, and experience of the respective physicians and some diffuse PCA can be missed by MRI^26^. Additionally, there are cancers missed by MRI with negative fusion biopsy, but cancer is detected in systematic cores, especially in smaller lesions or in cases where fusion of ultrasound and MRI is insufficient.

Furthermore, if a negative histopathology is already confirmed, our data suggest that follow-up mpMRI after 24 months seems sufficient not to miss csPCA and to reduce the number of unnecessary biopsies^13^. A possible explanation for the relatively longer time intervals in patients without PCA until a PI-RADS downgrade was observed may be inflammatory changes due to granulomatous or non-bacterial prostatitis or atypical hyperplasia. As long as these diffuse changes persist, they can potentially still mask PCA lesions and therefore PI-RADS category 3 is still justified^27–29^.

A recently published study by Washington SL et al. discussed the role of MRI based PSAD as a predictor for upgrade of the Gleason score under active surveillance^30^. This matches with our finding that PSAD significantly increased in patients with PCA between initial and follow-up mpMRI. In cases with PI-RADS 3 lesions and PSAD ≤ 0.2, the rate of ISUP grade ≥ 2 is vanishingly small^31^. PSAD alone without mpMRI showed poor performance in predicting csPCA in clinical routine over time^15^. Nonetheless, clinical parameters as PSAD seem to be a valuable tool in combination with mpMRI in uncertain cases to trigger or to postpone biopsy^32^.

Our study has limitations. First, this retrospective, single center study investigates a heterogeneous collective of patients who received follow-up mpMRI and subsequent biopsies at different time points. However, our study reflects a real-life scenario, and we were able to demonstrate significant changes even under these circumstances. We performed a systematic 12-core and additional MRI-targeted biopsy, taking two cores from each suspicious lesion. This is the standard in-house procedure and a common approach, also in larger studies. Nevertheless, there are different findings in the literature to perform targeted biopsy extracting more biopsy cores to reliably diagnose prostate cancer^33–35^. Even though other time intervals between the serial mpMRI, different from ours, may be thinkable, the intervals we used are based on guidelines for follow-up of patients under active surveillance and are widely used in clinical practice^13^. It is possible that different time intervals may have different outcomes. Further research on the optimal time interval is warranted.

In conclusion our results suggest that patients with PI-RADS category 3 may primarily receive follow-up mpMRI 12 to 24 months after the initial MRI scan instead of direct biopsy without missing csPCA. The overall number of csPCA in PI-RADS 3 lesions was very small and all patients with PI-RADS 3 lesions who harbored or developed csPCA over the course of the follow-up showed a PI-RADS upgrade in follow-up mpMRI in our cohort. This strategy may help to prevent overdiagnosis and overtreatment. In patients where the histopathologic results revealed no PCA, there was a significant downgrade in the PI-RADS category at follow-up mpMRI after 24 months. Therefore, patients with PI-RADS category 3 and negative biopsy do not seem to benefit from follow-up mpMRI earlier than after 24 months. In uncertain cases clinical parameters as PSAD may support clinical decision making.

## Supplementary Information

Supplementary Table 1.

## Data Availability

The datasets used and/or analyzed during the current study available from the corresponding author on reasonable request.

## Abbreviations

PCA: Prostate cancer
csPCA: Clinically significant prostate cancer (ISUP grade ≥ 2)
nsPCA: Non clinically significant prostate cancer (ISUP grade 1)
PI-RADS: Prostate Imaging Reporting and Data System
PSA: Prostate-specific antigen
PSAD: Prostate-specific antigen density
TRUS-GB: Transrectal ultrasound-guided prostate biopsy
IQR: Interquartile range
mpMRI: Multiparametic prostate MRI
