# Supplementary material for: Single center analysis of an advisable control interval for follow-up of patients with PI-RADS category 3 in multiparametric MRI of the prostate
Source: Sci Rep. 2022 Apr 25;12:6746. doi: 10.1038/s41598-022-10859-9 (PMC9038748; doi:10.1038/s41598-022-10859-9)
Supplement: Supplementary file 1 — Supplementary Table 1. [file 41598_2022_10859_MOESM1_ESM.docx]

|  |  | |  |  |  |  |
| --- | --- | --- | --- | --- | --- | --- |
| **ISUP**  **Grade Group** | | 1 | | **Baseline (n)** | **FU** |  |
|  |  |  |  | 15 | ↓ PI-RADS 2 2/15 |  |
|  |  |  |  |  | → PI-RADS 3  8/15 |  |
|  |  |  |  |  | ↑ PI-RADS 4  5/15 |  |
|  |  | 2 | | 3 | ↓ PI-RADS 2  0/3 |  |
|  |  |  |  |  | → PI-RADS 3  0/3 |  |
|  |  |  |  |  | ↑ PI-RADS 4  3/3 |  |
|  |  | 3 | | 1 | ↓ PI-RADS 2  0/1 |  |
|  |  |  |  |  | → PI-RADS 3  0/1 |  |
|  |  |  |  |  | ↑ PI-RADS 4  1/1 |  |

**Supp. Table 1:** Development of PI-RADS scores in patients with PCA and initial PI-RADs category 3 divided by ISUP group.

*FU = follow up; PCA = prostate cancer; PI-RADS = Prostate Imaging Reporting and Data System; ISUP = International Society of Urological Pathology (ISUP); ↓ = downgrade; → = stable; ↑ = upgrade*
